# Analytical sensitivity and specificity of a loop-mediated isothermal amplification (LAMP) kit prototype for detection of *Trypanosoma cruzi* DNA in human blood samples

**DOI:** 10.1371/journal.pntd.0005779

**Published:** 2017-07-20

**Authors:** Susana A. Besuschio, Mónica Llano Murcia, Alejandro F. Benatar, Severine Monnerat, Israel Cruz, Albert Picado, María de los Ángeles Curto, Yutaka Kubota, Diana P. Wehrendt, Paula Pavia, Yasuyoshi Mori, Concepción Puerta, Joseph M. Ndung'u, Alejandro G. Schijman

**Affiliations:** 1 Laboratorio de Biología Molecular de la Enfermedad de Chagas, Instituto de Investigaciones en Ingeniería Genética y Biología Molecular – Consejo Nacional de Investigaciones Científicas y Tecnológicas (INGEBI-CONICET), Buenos Aires Argentina; 2 Laboratorio de Parasitología Molecular, Facultad de Ciencias -Pontificia Universidad Javeriana, Bogotá, Colombia; 3 Foundation for Innovative New Diagnostics, Geneva, Switzerland; 4 Eiken Chemical Company, Tokyo, Japan; US Food and Drug Administration, UNITED STATES

## Abstract

This study aimed to assess analytical parameters of a prototype LAMP kit that was designed for detection of *Trypanosoma cruzi* DNA in human blood. The prototype is based on the amplification of the highly repetitive satellite sequence of *T*.*cruzi* in microtubes containing dried reagents on the inside of the caps. The reaction is carried out at 65°C during 40 minutes. Calcein allows direct detection of amplified products with the naked eye. Inclusivity and selectivity were tested in purified DNA from *Trypanosoma cruzi* stocks belonging to the six discrete typing units (DTUs), in DNA from other protozoan parasites and in human DNA. Analytical sensitivity was estimated in serial dilutions of DNA samples from Sylvio X10 (Tc I) and CL Brener (Tc VI) stocks, as well as from EDTA-treated or heparinized blood samples spiked with known amounts of cultured epimastigotes (CL Brener). LAMP sensitivity was compared after DNA extraction using commercial fiberglass columns or after “Boil & Spin” rapid preparation. Moreover, the same DNA and EDTA-blood spiked samples were subjected to standardized qPCR based on the satellite DNA sequence for comparative purposes. A panel of peripheral blood specimens belonging to Chagas disease patients, including acute, congenital, chronic and reactivated cases (N = 23), as well as seronegative controls (N = 10) were evaluated by LAMP in comparison to qPCR. LAMP was able to amplify DNAs from *T*. *cruzi* stocks representative of the six DTUs, whereas it did not amplify DNAs from *Leishmania* sp, *T*. *brucei* sp, *T*. *rangeli* KPN+ and KPN-, *P*. *falciparum* and non-infected human DNA. Analytical sensitivity was 1x10^-2^ fg/μL of both CL Brener and Sylvio X10 DNAs, whereas qPCR detected up to 1x 10^−1^ fg/μL of CL Brener DNA and 1 fg/μl of Sylvio X10 DNA. LAMP detected 1x10^-2^ parasite equivalents/mL in spiked EDTA blood and 1x10^-1^ par.eq/mL in spiked heparinized blood using fiberglass columns for DNA extraction, whereas qPCR detected 1x10^-2^ par.eq./mL in EDTA blood. Boil & Spin extraction allowed detection of 1x10^-2^ par.eq /mL in spiked EDTA blood and 1 par.eq/ml in heparinized blood. LAMP was able to detect *T*.*cruzi* infection in peripheral blood samples collected from well-characterised seropositive patients, including acute, congenital, chronic and reactivated Chagas disease. To our knowledge, this is the first report of a prototype LAMP kit with appropriate analytical sensitivity for diagnosis of Chagas disease patients, and potentially useful for monitoring treatment response.

## Author summary

*Trypanosoma cruzi*, a parasite transmitted to humans from hematophagous insects, causes Chagas Disease, a Neglected Tropical Disease with public health impact, affecting 7 million people in Latin America. Although mainly related to low income populations inhabiting rural environments, migrations have conveyed Chagas Disease to urban areas of endemic and non-endemic countries. It often presents non-specific symptoms, and direct, low cost microscopy-based diagnosis only detects acute infections, missing a high proportion of cases. Serology is the “gold standard” diagnostic technique for chronic stages and needs the concordance of at least two different assays to confirm infection. In this context, we aimed to evaluate the analytical sensitivity and specificity of a prototype kit based on a novel and rapid molecular biology reaction, named Loop mediated isothermal amplification (LAMP), using standardized Real Time PCR as a comparator. To our knowledge, this is the first LAMP prototype kit with an analytical performance appropriate for human diagnosis of Chagas disease and potentially useful for monitoring treatment response. Its simple handling using basic laboratory devices will enable point-of-care diagnosis and screening for congenital infection at birth as well as early detection of acute infections due to oral contamination.

## Introduction

Chagas disease, caused by the parasite *Trypanosoma cruzi*, remains a major concern in 21 endemic countries of Latin America, where infection is acquired mainly through the triatomine insect vector. Due to migration movements, it has spread over other continents, with 6 to 7 million people estimated to be infected. *T*. *cruzi* infection can also be transmitted by blood transfusion, the trans-placental route causing Congenital Chagas disease, oral contamination, organ transplantation and laboratory accident [1].

Two disease stages can be distinguished and the strategies for diagnosis are stage-dependent. Firstly, a short acute stage occurs with patent parasitemia that can be detected using conventional parasitological techniques, such as parasite microscopic observation in blood smears or microhematocrite, xenodiagnosis and hemoculture. However, these methods usually lack sensitivity and are operator dependent, and the last two mentioned techniques are cumbersome and their results can be acquired only several weeks after sample collection [2]. In a majority of acute cases, symptoms are not evident and thus the infection mostly goes undiagnosed; it enters in an indeterminate chronic period that may span life-time in around 70% of cases. In the remaining 30%, chronic stage leads to cardiomyopathy and/or digestive megasyndromes, causing death if untreated. As in the chronic phase, parasitemia is intermittent and low, diagnosis is largely made by serological tests. Due to the antigenic variability of the parasite, WHO’s guidelines recommend to perform at least two serological assays based on distinct antigen sets, which must agree for a conclusive diagnosis [1].

The different transmission modes, the disease phases and the high genetic variability of the parasite increase the difficulties of making diagnostic kits with most appropriate markers for the diverse Chagas disease epidemiological settings. Nucleic acid amplification strategies have opened new options to detect *T*. *cruzi* infection and evaluate anti-parasitic chemotherapy.

Diagnostic assays for Chagas disease need improvement. The development of diagnostic test for the following areas have been identified as a priority: acute phase, including oral and congenital transmission and monitoring of anti-parasitic treatment response [2, 3,]. The use of Loop-mediated isothermal amplification (LAMP) of DNA has been proposed as an outstanding approach to bridge some of these gaps [3]. LAMP is a platform developed by Eiken Chemical Company of Japan (http://www.eiken.co.jp/en). This technology detects known genes from different pathogens [4] [5] [6]. It has the following characteristics: (1) only one enzyme is used and the amplification reaction proceeds under isothermal conditions [7] [8]); (2) extremely high specificity because of the use of four primers recognizing six distinct regions on the target; (3) high amplification efficiency, with DNA being amplified 10^9^−10^10^ times within 15–60 minutes of incubation; and (4) it produces tremendous amounts of amplification product, making simple visual detection possible [8] [9]. Due to the above mentioned characteristics, LAMP can be performed in basic laboratories without the need for specialized infrastructure and it is appropriate for field applications and point-of-care diagnosis. Several of these kits are quite mature, such as the Tuberculosis and Malaria assays which are already commercialized Loopamp Assays. LAMP Assays—HUMAN Diagnostics Worldwide. Available: https://www.human.de/products/molecular-dx/isothermal-amplification/lamp-assays/)

A LAMP method for detection of *T*. *cruzi* DNA was previously designed based on the 18S ribosomal RNA (rRNA) gene and evaluated in DNA samples extracted from internal organs of triatomine vectors [10]. However, its low analytical sensitivity (100 fg per reaction tube) has not allowed its application to diagnosis of Chagas disease in humans.

LAMP tests based on amplification of multicopy repetitive sequences, such as the mobile element RIME of *T*. *brucei* [11] and the satellite DNA sequence of *T*. *vivax* [12] have been used to improve sensitivity. Accordingly, a novel LAMP assay based on the highly repetitive satellite DNA sequence of *T*.*cruzi* was developed to create a prototype kit for detection of this parasite in human blood. The present study aimed to assess the analytical performance of this kit prototype on DNA extracted from EDTA-treated as well as from heparinized human blood and explore its performance to detect *T*.*cruzi* DNA in blood samples from Chagas disease patients.

## Materials and methods

### Ethics statement

Informed written consent was obtained from all healthy donors and Chagas disease patients included in the study before blood collection, after permission of the IRB of the participating institutions, in agreement with argentine legislation in force (Blood Donation Law N° 22990, Res. N°1409/15)

### Participants

Peripheral blood samples from a total of 23 well-characterised Chagas disease patients and 10 seronegative controls were tested by LAMP and qPCR. Four clinical groups with *T*.*cruzi* infection were evaluated, namely Group CI: Samples from five newborns/neonates born to Chagas disease women; Group AI-TXRI: samples from three transplanted seronegative receptors of organs from a seropositive donor that acquired acute *T*.*cruzi* infection; Group CCD: samples from ten chronic Chagas disease patients; Group RCD: samples from five chronic Chagas disease patients undergoing clinical reactivation due to immunosuppression after organ transplantation. All cases and controls were admitted at Health Centers of Argentina.

### Parasitic strains

*T*. *cruzi* stocks belonging to the six discrete typing units (DTUs) [13] were used in the present study. Sylvio X10 (Tc I) and CL-Brener (Tc VI) are available at INGEBI since year 2009 and their identity is periodically assessed by multiplex Real Time PCR [14]. JG1 (Tc II), M6241 (Tc III) and M4167 (Tc IV) were kindly provided by Dr Constança Britto and Otacilio Moreira (Instituto Oswaldo Cruz, Rio de Janeiro, Brasil) and PAH179 (Tc V) by Dr Patricio Diosque (Instituto de Parasitología Experimental—IPE, University of Salta, Salta, Argentina). Strains were cultured using LIT medium and DNAs were purified using phenol-chloroform extraction followed by ethanol precipitation.

DNAs from *Leishmania* sp. and *T*.*rangeli* were kindly provided by Dr. Concepción Puerta from Pontificia Universidad javeriana—PUJ University, Bogotá, Colombia and DNA from *T*. *brucei* sp. and *P*. *falciparum* by Dr Yasuyoshi Mori from Eiken Chemical Company, Japan.

All DNAs were measured with microvolume UV-Vis spectrophotometer (Nanodrop 1000, Thermo Scientific, with nd_1000-v.3.8.1 software).

### DNA extraction for LAMP testing

DNA was extracted from EDTA blood and heparinized blood samples, using two different preparation procedures:

a) Blood based-DNA for LAMP was obtained from 200 μL of EDTA-blood using the High Pure PCR Template Preparation Kit with packed fiberglass filter tubes, cell lysis buffer and proteinase K (RAS extraction kit, Roche Applied Sciences, Mannheim, Germany) with one additional step prior to the addition of 100 μL of elution buffer, which consists of spinning the columns after the second wash to eliminate any traces of isopropanol and b) a Boil & Spin rapid procedure modified according to the type of blood sample: b1) Boil & Spin of EDTA-blood samples: 200 μL of blood were mixed with 200 μL of 0.5% SDS in double distilled water in a 1.5 mL microtube with screwcap and o-ring by vortex (10 seconds), then heated in a thermoblock at 95°C for 5 min. The tube content was spun down for 5 minutes at the maximum speed (13,300 rpm) on a bench top centrifuge. The supernatant was pipetted into a new labeled 1.5 mL flat cap microtube, ready to use immediately or stored at −20°C for up to 48 h, prior to use in the LAMP reaction, with only one freeze-thaw cycle before testing. b2) Boil & Spin of heparinized blood samples: 300 μL of blood and 15 μL of 10% SDS in double distilled water were thoroughly mixed in a 1.5 mL microtube with screwcap and o-ring by vortex (30 seconds). 100 μL of the solution were withdrawn, transferred to a new tube containing 400 μL of sterile water, then heated in a thermoblock at 90°C for 10 minutes. The tube content was spun down for 3 minutes at maximum speed (13,300 rpm). The supernatant was pipetted into a new labeled 1.5 mL flat cap microtube and the previous step was repeated once. The supernatant was used immediately for the LAMP reaction.

### DNA extraction for qPCR

Blood based-DNA for qPCR was obtained from 200 μL of EDTA-blood using the the RAS extraction kit as detailed for LAMP assays.

### Amplification procedure for Loopamp*Trypanosoma cruzi* prototype kit

This product is based on the nucleic acid amplification method, LAMP, developed by Eiken Chemical Co., Ltd. Japan, using as molecular target the repetitive satellite DNA sequence of *T*. *cruzi*. The primer sequences were designed after alignment analysis of selected database sequences to have a relatively well-conserved sequence recognized in all six described DTUs. The nucleotide sequence of the primers used in the study are not provided, since the assay is being developed into a commercial product. *T*. *cruzi* Loopamp kits can be obtained from Eiken (http://www.eiken.co.jp/en/inquiry.html.).

The reaction tube contains strand displacement Bst (*Bacillus stearothermophilus*) DNA polymerase, deoxynucleotide triphosphates (dATP, dCTP, dGTP and dTTP), calcein and *T*. *cruzi*-specific primers.

These reagents are in dried form on the inside of the cap of the reaction tube and are stable for one year at 30°C. A negative control (NC, distilled water) is provided in the kit. The positive control was 1fg/μL of DNA from CL-Brener stock (Tc VI).

The LAMP reaction was performed as follows: 30 μL of sample DNA extract was dispensed into each LAMP tube, and the cap was closed. Each tube was flicked down to collect the solution at the bottom and placed upside down during two minutes to reconstitute the dried reagent. It was inverted five times to mix the content followed by a spin down.

Incubation of the reaction was carried out at 65°C for 40 minutes for isothermal amplification, followed by a step at 80°C for five minutes for enzyme inactivation using different devices: 1) Rotor Gene 6000 thermocycler (Corbett Life Science, Cambridgeshire, UK); Perkin-Elmer 9600 (Thermofisher, USA) and Genie III Fluorimeter Instrument (Optigene, Horsham, West Sussex, UK).The results of amplification were visualized using different strategies: i) Real-time fluorescence data was obtained on the Rotor Gene 6000 FAM channel (excitation at 470 nm and detection at 510 nm, auto-optimisated gain level -2, 33 after 40 cycles of 60 seconds each at 65°C to achieve an isothermal reaction followed by a hold on 80°C during 5 minutes) ii) Genie III Instrument (excitation at 470 nm, detection at 510 nm, low gain level 4, specific for calcein; the run profile settings for amplification were 65°C, 40 min. To end reaction, the anneal settings starts at 98°C with a ramp rate of 0,06°C/second to reach 80°C during 5 minutes in order to inactivate the enzyme iii) visualization of fluorescence by the naked eye iv) in some cases, 1.2% agarose gel electrophoresis with TAE (Tris-acetate with EDTA), ethidium bromide and GelPilot loading dye 5x, final dilution 1x, run at 80 V (Qiagen, Hilden, Germany) was carried out to corroborate the typical ladder profile of multiple bands of LAMP products [15].

### Amplification using Real Time quantitative PCR (qPCR)

The LAMP method was compared to a standardized qPCR assay that consisted of a duplex qPCR with TaqMan probes targeted to *T*. *cruzi* satellite DNA and Internal Amplification control (IAC), as described by Duffy and coauthors [16] on the basis of the *T*.*cruzi* primer and probe sequences published by Piron and coauthors [17], and validated following international guidelines [16,18]. The qPCR reactions were carried out in duplicates with 5 μL of eluted DNA in a final volume of 20 μL containing 2X FastStart Universal Probe Master Mix (Roche Diagnostics GmbHCorp., Mannheim, Germany), 10 μM primers *cruzi* 1 and *cruzi* 2 and probe *cruzi* 3 [19]; 5 μM primer IAC FW, primer IAC Rv and probe IAC-Tq [14]. Cycling conditions were a first step of 10 minutes at 95°C followed by 40 cycles at 95°C for 15 seconds and 58°C for 1 minute. The amplifications were carried out in an ABI7500 (Applied Biosystems, Foster City, CA, USA) or a Rotor-Gene 6000 (Corbett Life Science, Cambridgeshire, United Kingdom) thermocycler equipments. All reactions included a strong positive control (SPC): 10 fg/μL of CL Brener DNA and a weak positive control (WPC): 1 fg/μL of CL-Brener DNA.

### Analytical validation of LAMP Assay

#### Selectivity

Inclusivity was tested in serial dilutions of purified DNAs from *T*. *cruzi* stocks belonging to the six DTUs, namely Sylvio X10 (Tc I), JG1 (Tc II), M6241 (Tc III), M4167 (Tc IV), PAH179 (Tc V) and CL-Brener (Tc VI).

Exclusivity was tested in purified DNAs from *T*. *rangeli KPN+ and KPN- stocks*, *T*.*b*. *brucei*, *T*.*b*. *gambiense*, *T*.*b*. *rhodesiense*, *Leishmania amazonensis*, *L*. *donovani*, *L*.*infantum*, *L*. *infantum chagasi*, *L*. *major and L*. *mexicana*, and *Plasmodium falciparum*.

Human DNA samples from blood collected from 5 individuals who were seronegative for *T*. *cruzi* were included as specificity controls. DNA was obtained using commercial RAS columns, as indicated above. Human DNA was tested without prior quantification in spectrophotometer, because it constitutes part of the matrix of blood samples. LAMP reactions were done in triplicate.

#### Analytical sensitivity in purified DNA

To evaluate the analytical sensitivity of LAMP, three serial ten-fold dilutions spanning 1 x 10^3^ to 1 x 10^−3^ fg /μL were prepared from DNA aliquots of Sylvio X10 (Tc I) and CL Brener stocks (Tc VI) from an already-existing collection from our laboratory. These strains were selected because they present the highest difference in copy number of Satellite DNA repeats [19]. LAMP reactions were done in triplicates from each DNA aliquot. The four most diluted samples were also tested by qPCR for comparative purposes; three different DNA aliquots from the same DNA stock were assayed in duplicates for each concentration.

#### Analytical sensitivity in spiked human blood samples

Analytical sensitivity using spiked blood samples was carried out in EDTA-blood and heparinized blood samples extracted using RAS extraction kit in order to explore the effects of the matrix from human blood samples. Whole EDTA or heparinized blood collected from non-infected donors was spiked with ten-fold serial dilutions of CL Brener cultured parasites spanning concentrations of 1 x 10^3^ to 1 x 10^−3^ parasite equivalents/mL (par.eq./mL). The term parasite equivalent means the equivalent genomic content of one parasite cell, as the blood samples are spiked with parasite epimastigotes manually counted using a Neubauer chamber. This stock was the one selected for this measurement because it has been previously used to evaluate analytical sensitivity of the standardized and validated qPCR procedure, used as a reference in this work [16].

#### Evaluation of LAMP in clinical samples

A panel of peripheral blood specimens belonging to well-characterized Chagas disease patients, including acute, congenital, chronic and reactivated cases (N = 23) and seronegative controls (N = 10) were evaluated by LAMP in comparison to qPCR.

All samples were obtained in EDTA-tubes (BD vacutainer tubes, Becton Dickinson, USA) and processed using the RAS kit for DNA purification. A volume of 30 μL of DNA eluate was used for LAMP. All samples were also run by qPCR for comparative purposes; 5 μL of DNA eluate was used.

## Results

### Selectivity

#### Inclusivity

**Fig 1:**
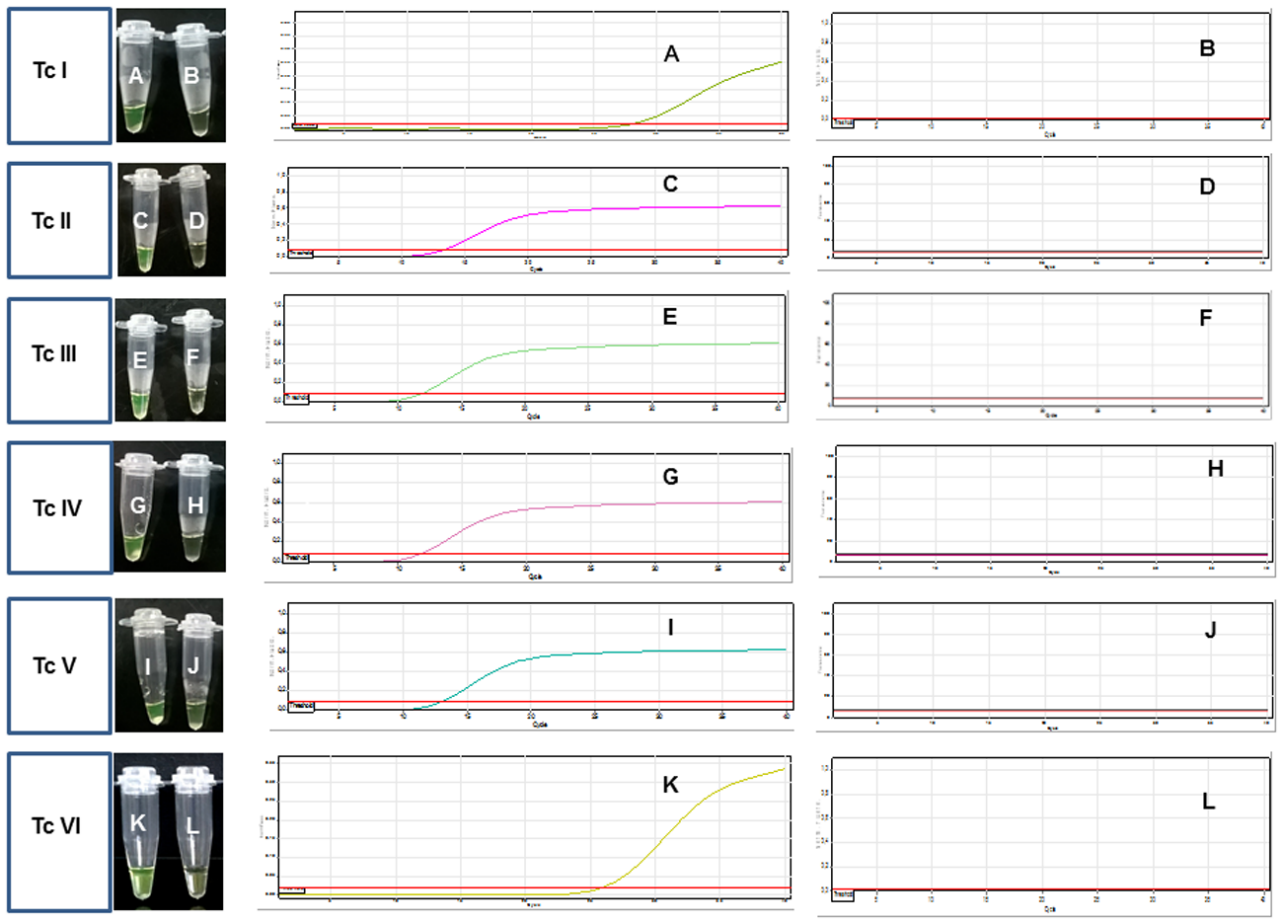
Inclusivity of LAMP assay tested in purified DNA samples from *T*. *cruzi* strains representative of the different discrete type units. Left panels: Visualization by the naked eye of *T*. *cruzi* DNA stocks representative of different DTUs. Tc I (A: 1.0 x10^-2^ fg/test; B: 1.0 x10^-3^ fg/test); Tc II (C: 2.5 fg/test; D: 2.5x10^-1^ fg/test); Tc III (E: 7.5 x 10^−2^ fg/test; F: 7.5 x 10^−3^ fg/test); Tc IV (G: 5.0 x 10^−1^ fg/test, H: 5.0 x 10^−2^ fg/test); Tc V (I: 1.5 x 10^−1^ fg/test; J: 1.5 x 10^−2^ fg/test); Tc VI (K: 1.0 x 10^−1^ fg/test; L. 1.0 x 10^−2^ fg/test) Right panels: Amplification plots obtained in the LAMP reaction after analyzing the samples in a Rotor Gene 3000 thermocycler. Y axis represents fluorescence and x axis represents Cts (Threshold cycles). Only the highest dilution giving amplification and the next dilution giving non detectable results are shown.

The LAMP assay was able to detect DNA from *T*. *cruzi* stocks representative of DTUs I to VI. The *T*. *cruzi* stocks that showed detectable LAMP results at the lowest DNA concentrations were M6241 (Tc III, 7.5 x 10^-2^ fg/test) and M4167 (Tc IV, 5.0 x 10^-2^ fg/test) in one of both tested duplicates and the stock with lowest sensitivity was JG1 (Tc II, 2.5 fg/test) in both replicates (Fig 1 and Table 1).

**Table 1:** INCLUSIVITY in *Trypanosoma cruzi* strains representative of different DTUs (fg/test).

Tc I (Sylvio X10)	1.0 x 10^−1^	1.0 x 10^−2^	1.0 x 10^−3^	1.0 x 10^−4^
Naked eye detection	+/-	+ /-	-/-	-/-
Real Time mean Ct^×^	23.25	27.8	NDt/NDt	NDt/NDt
Tc II (JG1)	2.5	2.5 x 10^−1^	2.5 x 10^−2^	2.5 x 10^−3^
Naked eye detection	+/+	-/-	-/-	-/-
Real Time mean Ct	13.47	NDt/NDt	NDt/NDt	NDt/NDt
Tc III (M6241)	7.5 x 10−1	7.5 x 10−2	7.5 x 10−3	7.5 x 10−4
Naked eye detection	+/+	+/-	-/-	-/-
Real Time mean Ct	8.68	11.81/NDt	NDt/NDt	NDt/NDt
Tc IV (M4167)	5.0	5.0 x 10−1	5.0 x 10−2	5.0 x 10−3
Naked eye detection	+/+	+/+	+/-	-/-
Real Time mean Ct	8.66	11.88	12.74/NDt	NDt/NDt
Tc V (PAH179)	1.5	1.5 x 10^−1^	1.5 x 10^−2^	1.5 x 10^−3^
Naked eye detection	+/+	+/+	-/-	-/-
Real Time mean Ct	10.20	12.98	NDt/NDt	NDt/NDt
Tc VI (CL-Brener)	1.0	1.0 x 10^−1^	1.0 x 10^−2^	1.0 x 10^−3^
Naked eye detection	+/-	+/-	-/-	-/-
Real Time mean Ct	12.68	25.24	NDt/NDt	NDt/NDt

. NDt: Non detectable. ^X^ When only one replicate was LAMP positive, the Ct of that sample was reported.

#### Exclusivity

**Fig 2:**
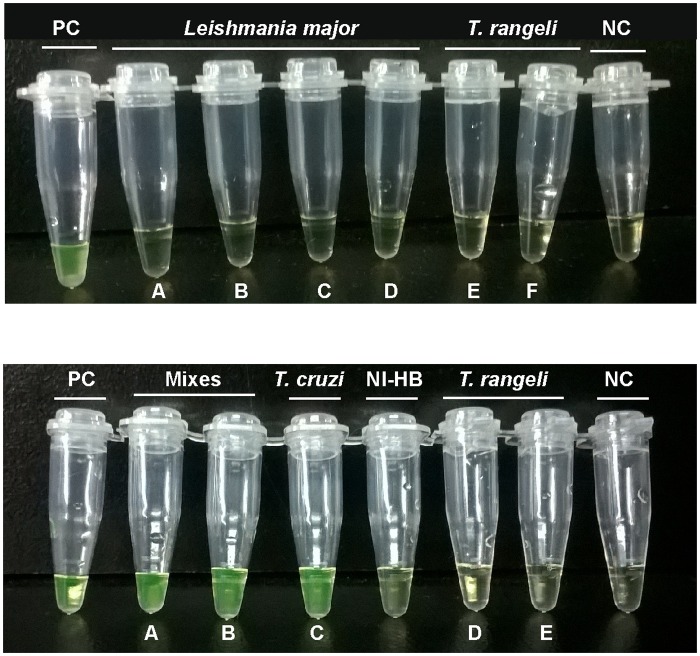
Exclusivity of LAMP assay tested in purified DNA samples from *T*. *rangeli*, *Leishmania major* stocks and non-infected human DNA. Top Panel. (A): *Leishmania major* DNA was tested at 1.0 x 10^1^fg/ μL; (B) 1.0 x .10^2^ fg/ μL; (C) 1.0 x 10^3^ fg/ μL; (D) 1.0 x. 10^4^ fg/ μL. *T*. *rangeli* DNA was tested (E) at 1.0 x 10^4^ fg/ μL and (F) 1.0 x .10^3^ fg/μL. Bottom Panel. (A): Mixture containing equal volumes of *T*. *cruzi* and *Leishmania major* DNA at 1.0 x 10^4^ fg/μL. (B): Mixture containing equal volumes of *T*. *cruzi* and *T*. *rangeli* DNA at 1.0 x 10^4^ fg/μL. (C): *T*. *cruzi* DNA tested at 10 fg/μL. (D) and (E): *T*. *rangeli* DNA tested at 10 and 100 fg/μL, respectively. NIHB: Non-infected human DNA. PC: Positive Control. NC: Negative Control.

No LAMP products were obtained when up to 30 ng/test of DNA samples from *P*. *falciparum*, different species of *Leishmania (L*.*major; L*. *amazonensis*, *L*.*chagasi*,*L*.*donovani*, *L*. *infantum*, *L*.*guyanensis and L*.*mexicana)*, *T*. *brucei subspecies (T*.*brucei brucei*, *T*.*b*.*gambiense*, *T*.*b*. *rhodesiense)* and *T*. *rangeli* (KPN + and KPN—stocks) were tested (examples in Fig 2A).

Non-infected human DNA gave negative LAMP results (Fig 2B). In order to confirm that the negative LAMP results were not due to reaction inhibition by the high DNA concentrations tested, mixtures of *T*. *cruzi* DNAs with *Leishmania major or T*. *rangeli* KPN+ were assayed (Fig 2B). LAMP results were positive, showing that there was no inhibitory effect by the DNA concentrations of *Leishmania major* or *T*.*rangeli* KPN+ (Fig 2B).

The standardized qPCR assay, used as comparator, was carried out on DNA samples from *Leishmania sp*, *T*. *rangeli* KPN+ and non-infected human DNA dilutions, and negative findings were observed, in agreement with LAMP results (S1 Fig).

### Analytical sensitivity of LAMP assay

**Fig 3:**
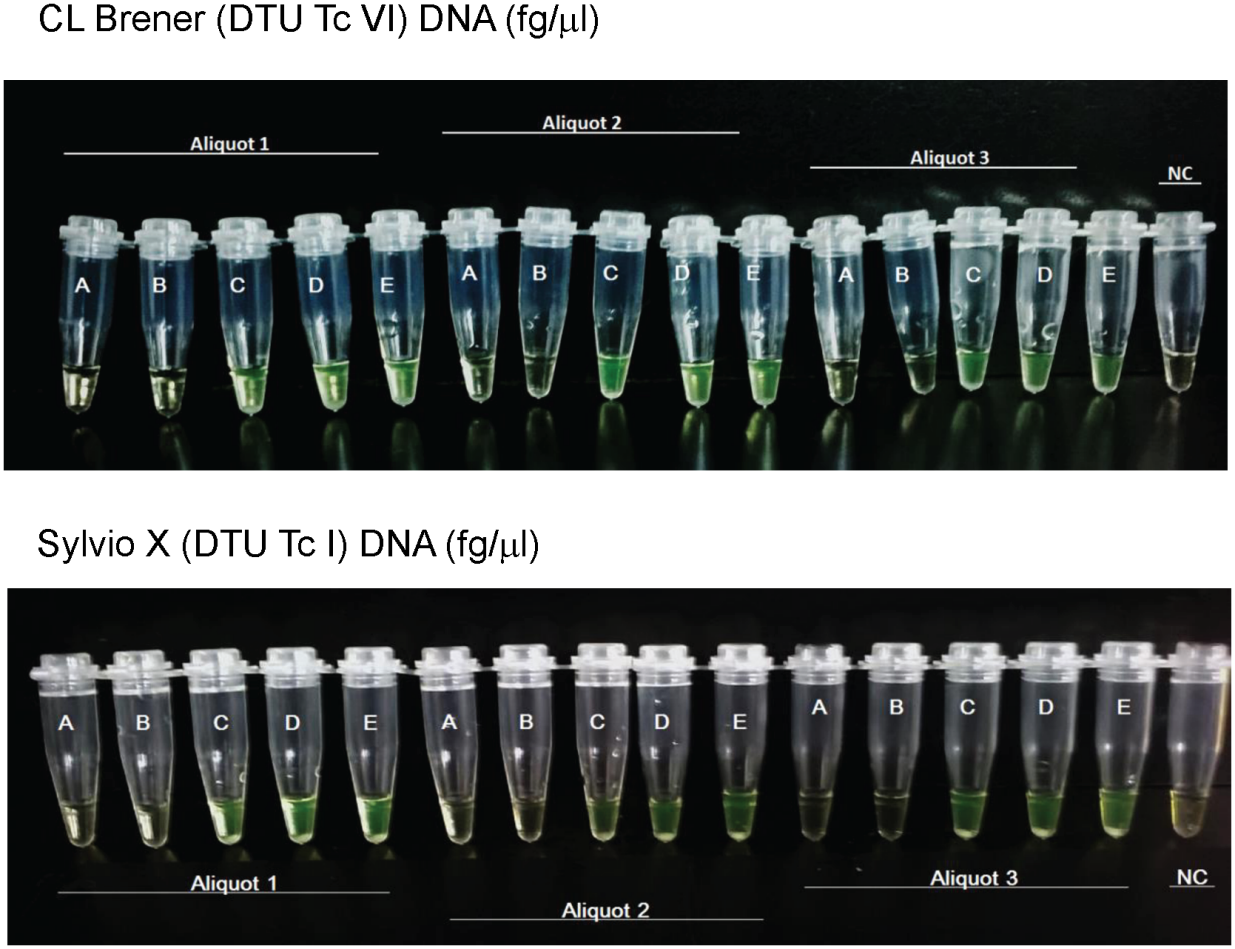
Analytical sensitivity of LAMP assay. Top panel: Fluorescence observed with the naked eye from serial dilutions obtained from 3 different aliquots of DNA from *CL* Brener stock (DTU VI). Bottom panel: Fluorescence observed with the naked eye from serial dilutions obtained from 3 different aliquots of DNA from Sylvio X10 stock.The aliquots were expressed in fg/μL. A: 0; B: 1.0 x 10^−3^; C: 1.0 x 10^−2^; D: 1.0 x 10^−1^, E: 1. NC: Non template control.

Analytical sensitivity was estimated on ten-fold serial dilutions done in triplicate from three independent DNA samples of Sylvio X10 (Tc 1) and CL Brener (Tc VI) stocks (Fig 3).

The prototype LAMP kit detected *T*.*cruzi* DNA at concentrations ≥ 1 x 10^−2^ fg/μL (0.3 fg per test) in both CL Brener and Sylvio X10 stocks. DNA samples spanning 1 fg/μL to 1 x 10^−3^ fg/μL were also assayed by qPCR for comparative purposes (S2 and S3 Figs). The qPCR detected up to 1 x 10^−1^ fg/μL of CL Brener DNA (S2 Fig) and 1 fg/μL of Sylvio X10 DNA, whereas 1 x 10^-1^fg/μL of the latter was detectable in only one aliquot (S3 Fig, aliquot A1).

### Analytical sensitivity and specificity of LAMP assay in spiked blood samples

The analytical sensitivity of LAMP was tested in EDTA and heparinized-blood samples spiked with known parasite loads (par.eq./mL).

**Fig 4:**
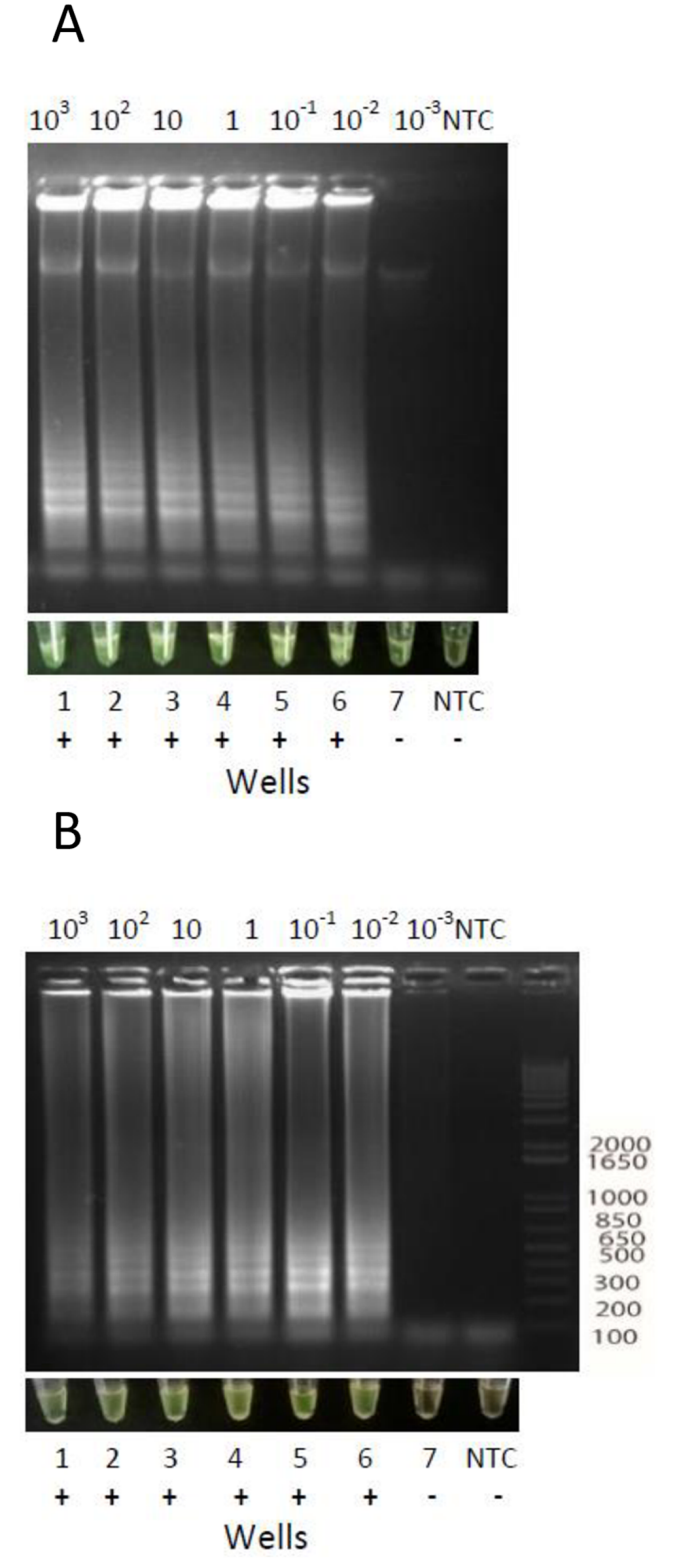
Analytical sensitivity of LAMP assay in spiked EDTA blood samples. EDTA blood samples spiked with different quantities of purified *T*. *cruzi* DNA were processed using RAS columns or the Boil & Spin method. (A) EDTA blood processed using RAS columns. (B): EDTA blood processed by Boil & Spin method. Upper panels: LAMP reaction products analyzed by electrophoresis in 1.2% agarose gels and stained with ethidium bromide. Bottom panels: pictures of LAMP reaction products visualized by the naked eye.

EDTA blood samples spiked with CL Brener DNA showed positive LAMP results when using the RAS extraction kit (6 x 10^−4^ par.eq. per test; Fig 4A) and the Boil & Spin preparation method (3 x 10^−4^ par.eq. per test; Fig 4B).

The qPCR was carried out in spiked EDTA blood samples following the standardized procedure, which gave a sensitivity of 1 x 10^−2^ par.eq./mL (S1 Table)

#### Heparinized-blood

**Fig 5:**
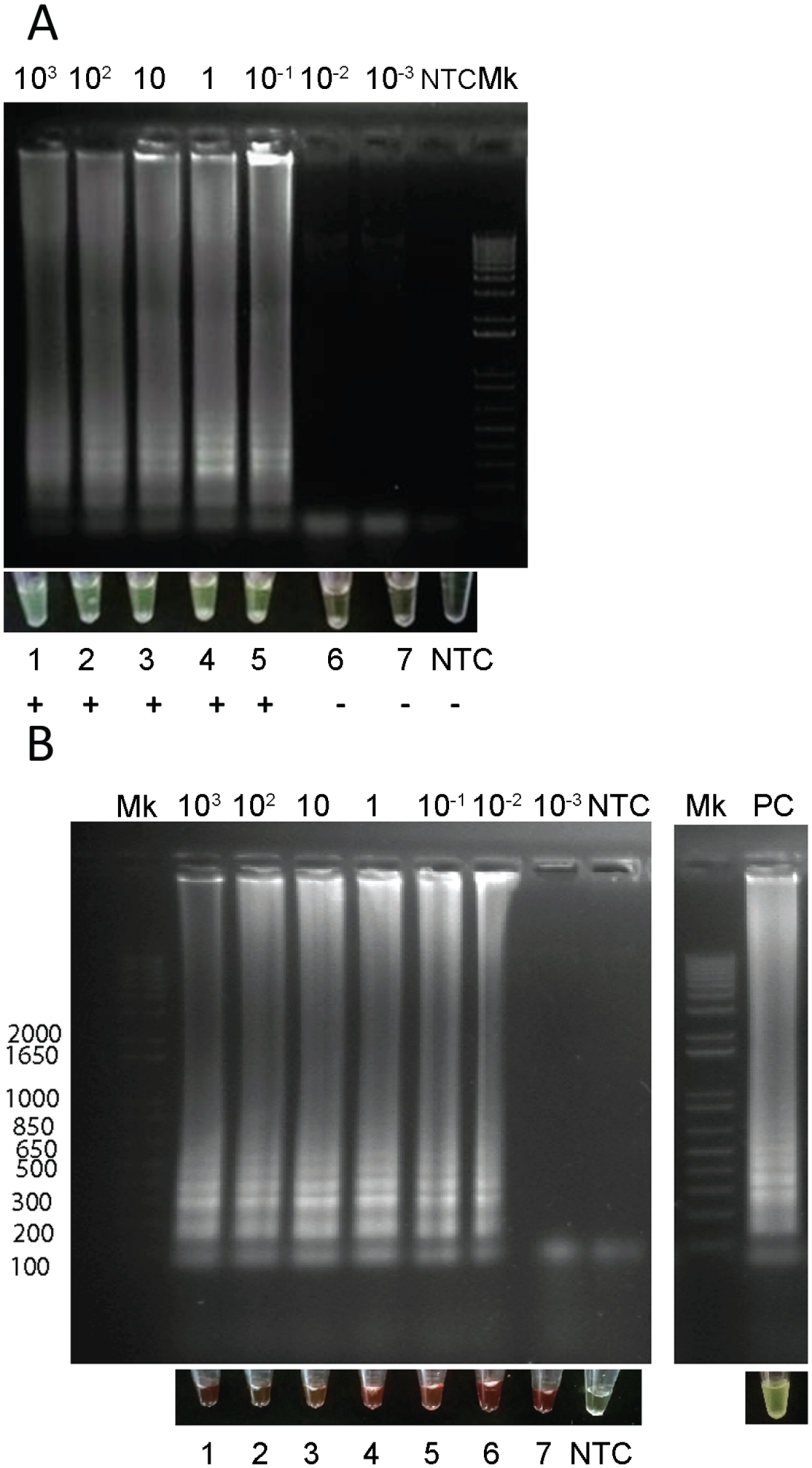
Analytical sensitivity of LAMP assay done in spiked heparinized blood samples extracted using commercial columns or Boil & Spin procedure. A. Heparinized blood processed by RAS columns. B. Heparinized blood processed by Boil & Spin method. For each DNA extraction method, samples were visualized with the naked eye and reaction products were observed after 1.2% agarose gel electrophoresis and ethidium bromide staining.

Spiked heparinized blood samples processed by RAS extraction kit detected up to 1 x 10^−1^ CL Brener par.eq/mL (6 x 10^−3^ par.eq per test) (Fig 5A).

When processed by Boil & Spin, reactions starting from 30 μLs of DNA preparation presented a reddish appearance that precluded visualization by the naked eye, whereas agarose gels showed the occurrence of amplification (Fig 5B).

Only after performing serial ten-fold dilutions of the Boil & Spin DNA preparation, direct visualization was possible when at least 1:100 dilutions were used, between the range 10^2^ to 1 par.eq./mL (S4 Fig).

### Evaluation of Clinical Specimens using LAMP and qPCR

A panel of peripheral blood specimens belonging to Chagas disease patients (N = 23), as well as seronegative controls (N = 10) were evaluated by LAMP in comparison to qPCR. LAMP allowed detection of *T*.*cruzi* DNA in all congenital Chagas disease (CI), seronegative receptors of organs from seropositive donors with acute infection (AI-TXRID) and reactivated Chagas disease (RCD) patients, in agreement with qPCR positivity (Table 2). However, in a panel of samples from ten chronic Chagas disease patients (CCD), LAMP detected *T*.*cruzi* DNA in four, whereas qPCR was positive in three, suggesting higher sensitivity of LAMP with respect to qPCR. LAMP gave negative results in samples from ten seronegative patients in agreement with qPCR results (Table 2, NI).

**Table 2:** Clinical specimens results from LAMP test and qPCR as a comparator.

Patient ID	Clinical Group	q PCR RESULT	Ct (average)	Parasitic load (par.eq./mL)	LAMP RESULT
1	CI1	Positive	25.05	53.05	Positive
2	CI2	Positive	24.46	75.31	Positive
3	CI3	Positive	25.44	37.47	Positive
4	CI4	Positive	27.44	12.90	Positive
5	CI5	Positive	28.63	5.26	Positive
6	AI-TXRID 1	Positive	18.74	2939.50	Positive
7	AI-TXRID 2	Positive	27.20	11.50	Positive
8	AI-TXRID 3	Positive	18.25	4060.50	Positive
9	CCD 1	Positive	27.45	10.50	Positive
10	CCD2	Positive	29.01	3.50	Positive
11	CCD3	Positive	26.20	22.00	Positive
12	CCD4	Negative	ND	ND	Positive
13	CCD5	Negative	ND	ND	Negative
14	CCD6	Negative	ND	ND	Negative
15	CCD7	Negative	ND	ND	Negative
16	CCD8	Negative	ND	ND	Negative
17	CCD9	Negative	ND	ND	Negative
18	CCD10	Negative	ND	ND	Negative
19	RCD 1	Positive	27.85	7.50	Positive
20	RCD 2	Positive	22.83	201.50	Positive
21	RCD 3	Positive	24.45	70.00	Positive
22	RCD 4	Positive	29.34	2.50	Positive
23	RCD 5	Positive	28.04	7.00	Positive
24	NI 1	Negative	ND	ND	Negative
25	NI 2	Negative	ND	ND	Negative
26	NI 3	Negative	ND	ND	Negative
27	NI 4	Negative	ND	ND	Negative
28	NI 5	Negative	ND	ND	Negative
29	NI 6	Negative	ND	ND	Negative
30	NI 7	Negative	ND	ND	Negative
31	NI 8	Negative	ND	ND	Negative
32	NI 9	Negative	ND	ND	Negative
33	NI 10	Negative	ND	ND	Negative

**CI** Congenital infection; **AI-TXRID** Acute infection of Transplanted recipient from infected donor; **CCD** Chronic Chagas Disease; **RCD** Reactivated Chagas Disease; **NI** Non infected patient.

**Fig 6:**
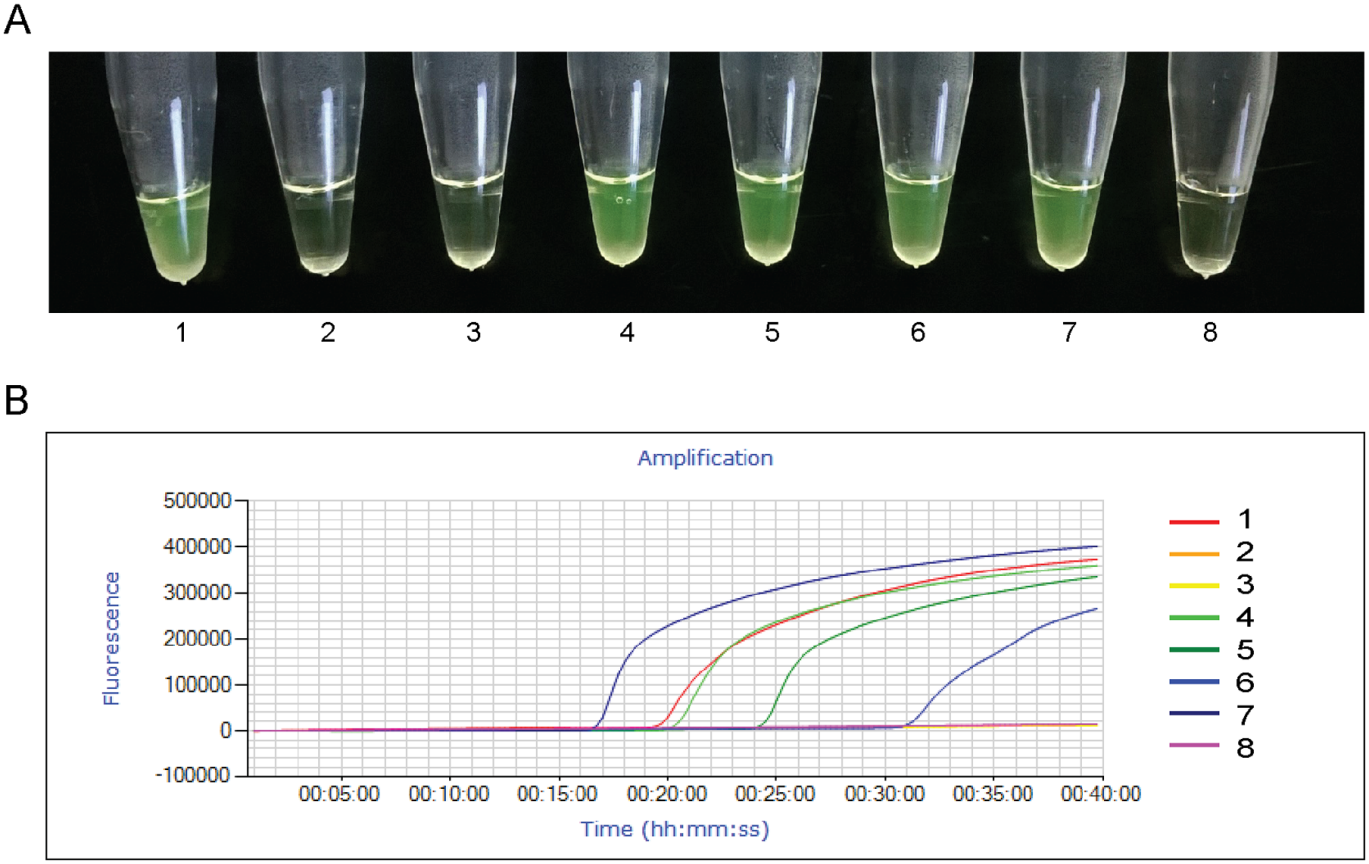
Evaluation of clinical specimens using LAMP assay. A. Visualization by Naked Eye: 1. positive control; 2. CCD6 Chronic Chagas Disease (case 6); 3. NI8: non-infected patient, (case 8); 4. AI-TxRID 2: acute infection of transplanted recipient from infected donor (case 2); 5. RCD 1: reactivated Chagas disease (case 1); 6. CCD1: chronic Chagas disease 1 (case 1); 7. CI 4: congenital Chagas disease (case 4); 8. negative control. B. Detection of LAMP reaction using Genie III Fluorimeter. 1: positive control; 2 to 7: clinical specimens indicated in A; 7: Negative control. The Y Axis denotes Fluorescence and X axis denotes Tt (time when fluorescence passes the threshold).

Examples of the tested cases are illustrated in Fig 6 (LAMP results) and S5 Fig (qPCR results).

## Discussion

Clinical molecular diagnosis of Chagas disease is important for: (i) early diagnosis of congenital transmission in newborns when presence of maternal anti-*T*. *cruzi* antibodies may deliver false positive results [20], (ii) early detection of infection in transplant receptors of organs from a Chagas positive donor [21], (iii) monitoring of parasite reactivation in chronically infected patients immune-suppressed due to organ transplantation [22] or AIDS [23], and (iv) the evaluation of new treatments in clinical trials, because detection of serological negative conversion in seropositive patients showing a favorable treatment outcome is impractical from a study time perspective [24] [25].

This work aimed to assess the performance of a LAMP kit prototype targeted to satellite *T*. *cruzi* DNA, a highly repetitive and conserved sequence in all characterized *T*.*cruzi* strains.

The prototype kit has the advantage of using reagents in dried form on the inside of the cap of the reaction tubes. Moreover, the use of calcein allows direct visualization of amplification by the naked eye. Calcein is included in the reaction tubes in a quenched state, bound to manganese ions. Once the LAMP reaction starts, pyrophosphate ions that are generated bind to the manganese ions, releasing calcein and generating fluorescent light. Furthermore, the presence of magnesium ions in the buffer system enhance calcein fluorescence [9].

Analytical sensitivity and specificity of the kit prototype was carried out using purified DNA from different parasite stocks representative of the six *T*. *cruzi* DTUs, as well as from human blood samples anti-coagulated with EDTA or with heparin and spiked with known quantities of *T*. *cruzi* cells. Furthermore, the prototype was evaluated in a blind panel of peripheral blood samples from well characterized Chagas disease patients at different stages and with different clinical manifestations, as well as seronegative donors as non-infected controls (Table 2 and S5 Fig).

The LAMP kit was able to amplify DNA from all tested *T*.*cruzi* stocks by the naked eye and by Real Time detection of fluorescence in a Rotor Gene Corbett Thermocycler (Table 1).

Analytical sensitivity was calculated for Tc I and Tc VI stocks, among other parasite strains, and was higher than the analytical sensitivity observed with the same stocks using standardized qPCR based on the same target [16]. The kit does not intend to discriminate between DTUs. Variations in Cts among *T*.*cruzi* stocks can be explained due to the heterogeneity in copy numbers of satellite repeats [19], however sensitivity was high for all tested strains. Indeed, satellite DNA has been successful as a molecular target for LAMP assays to detect infections by *T*. *vivax* [12] and to implement a direct dried blood based diagnostic tool for Human African trypanosomiasis [11] [26].

LAMP was specific for *T*. *cruzi* DNA since negative findings were obtained even with high concentrations (up to 1 pg/μL) of DNAs from *T*. *rangeli (KPN+ and KPN- stocks)*, *P*. *falciparum*, several *Leishmania* species, and *T*. *brucei* sub-species.

The use of EDTA as anticoagulant proved to be suitable in our study, although it has not been recommended for LAMP, due to the fact that EDTA competes for manganese ions with the pyrophosphate ions generated once the reaction starts [27]. Nevertheless, LAMP analysis carried out using DNA obtained from EDTA-blood samples has also been reported [26]. Since an easy and quick detection is desirable for application of a LAMP prototype in point-of-care diagnosis, EDTA-blood samples are appropriate since they are routinely withdrawn in most health care centers; i.e. for hemogram reports. In fact, we detected up to 1 x 10^−2^ par. eq./mL in extracts from spiked EDTA blood samples using fiberglass commercial columns as well as after Boil & Spin, in accordance to the sensitivity detected by qPCR.

In contrast, when LAMP experiments were performed in heparinized spiked blood using RAS columns, sensitivity was one order below. Moreover, LAMP results obtained using 30 μL of Boil & Spin DNA preparations from heparinized blood could only be detected after agarose gel electrophoresis, whereas observation of fluorescence with the naked eye was not possible: reddish appearance of the contents of tubes hampered visualization (Fig 5B). Direct visualization was possible only after diluting the DNA extracts at least 1:100 times. However, the analytical sensitivity in those diluted samples was poor in comparison to the detection limit achieved using the other methods; it was only 1 par.eq./mL (S4 Fig). Consequently, the Boil & Spin procedure used in our study needs to be revised for improvement. It performed well in other Loopamp kits, such as those developed for Pan/ *P*. *falciparum* (27, 28) and *Trypanosoma brucei* detection (Standard operating procedures for the Loopamp Trypanosoma Available: https://www.finddx.org/wp-content/uploads/2016/06/HAT-LAMP-SOP_13JUN16.pdf).

The analytical sensitivity of this LAMP assay was superior to that previously obtained using a LAMP procedure based on the *18S rDNA* gene [10]. The mentioned study was done using DNA from Tulahuen strain (Tc VI), and analytical sensitivity was 100 fg per test, using a Real-time turbidimeter, detection under UV light and direct visualization by the naked eye after 60 minutes of incubation.

To our knowledge, this is the first report of a LAMP kit with similar analytical sensitivity than Real Time PCR. It was validated using the same *T*.*cruzi* strains [16, 28] and using different methods for visualization of amplification. Our data provide evidence of the usefulness of this LAMP kit for molecular diagnosis of Chagas disease. Prospective analysis of clinical specimens will allow establish its performance in different epidemiological and clinical scenarios, such as early diagnosis of congenital infection, POC detection of acute infections due to oral transmission or in seronegative recipients of organs from seropositive donors, early detection of reactivation due to immunosuppression due to organ transplantation or AIDS [3,20,21,22,23,29]. Furthermore, the persistence of positive LAMP results in patients under etiological treatment could be useful to assess treatment failure [3, 24, 25, 30, 31,32].

## Supporting information

S1 Fig Analytical specificity of qPCR tested in purified DNA from *Leishmania major*, *T*.*rangeli* and non-infected human DNA. Y axis indicates normalized fluorescence and X axis denotes Cycles. SPC: Strong positive control: 10 fg/μL of *T*.*cruzi* DNA; WPC: weak positive control: 1 fg/μL of *T*.*cruzi* DNA.
(TIF)

S2 Fig Analytical sensitivity of qPCR for CL Brener stock. qPCR assays were performed using CL Brener DNA at concentrations ranging from 1 x 10^−3^ to 1 fg/μL. SPC: Strong positive control: 10 fg/μL of *T*.*cruzi* DNA; WPC: weak positive control: 1 fg/μL of *T*.*cruzi* DNA. Y axis indicates normalized fluorescence and X axis denotes Cycles.
(TIF)

S3 Fig Analytical sensitivity of qPCR for Sylvio X10 stock. qPCR assays performed using the same concentrations of Sylvio X10 DNA tested by LAMP. SPC: Strong positive control: 10 fg/μL of *T*.*cruzi* DNA; WPC: weak positive control: 1 fg/μL of *T*.*cruzi* DNA. Y axis indicates normalized fluorescence and X axis denotes cycles.
(TIF)

S4 Fig LAMP assay performed in *T. cruzi* DNA extracted from spiked heparinized blood processed by the Boil & Spin method". LAMP test of serial ten-fold dilutions of *T*.*cruzi* DNA extracted from a 10^4^ par.eq./mL spiked heparinized blood sample using Boil & Spin method. NTC: Negative Control (distilled water); A, 1:10 dilution (10 ^3^ par.eq/mL); B, 1:100 dilution (10 ^2^ par.eq./mL); C, 1:1000 dilution(10 par.eq./mL); D,1:10^4^ dilution (1 par.eq./mL); 1:10^5^ dilution (E: 10^-1^ par.eq./mL); NIHB: Non Infected Human Blood; PC: Positive C.
(TIF)

S5 Fig Evaluation of clinical specimens using qPCR. NTC: non template control; SPC: strong positive control; WPC: weak positive control. Clinical samples indicated in Fig 6, panel A. The Y axis denotes fluorescence and the X axis denotes Cts. control.
(TIF)

S1 Table Analytical sensitivity of qPCR in EDTA-blood samples spiked with CL-Brener cells. (DOCX)
