# Supplementary figures and images for: Analytical sensitivity and specificity of a loop-mediated isothermal amplification (LAMP) kit prototype for detection of Trypanosoma cruzi DNA in human blood samples
Source: PLoS Negl Trop Dis. 2017 Jul 20;11(7):e0005779. doi: 10.1371/journal.pntd.0005779 (PMC5544240; doi:10.1371/journal.pntd.0005779)

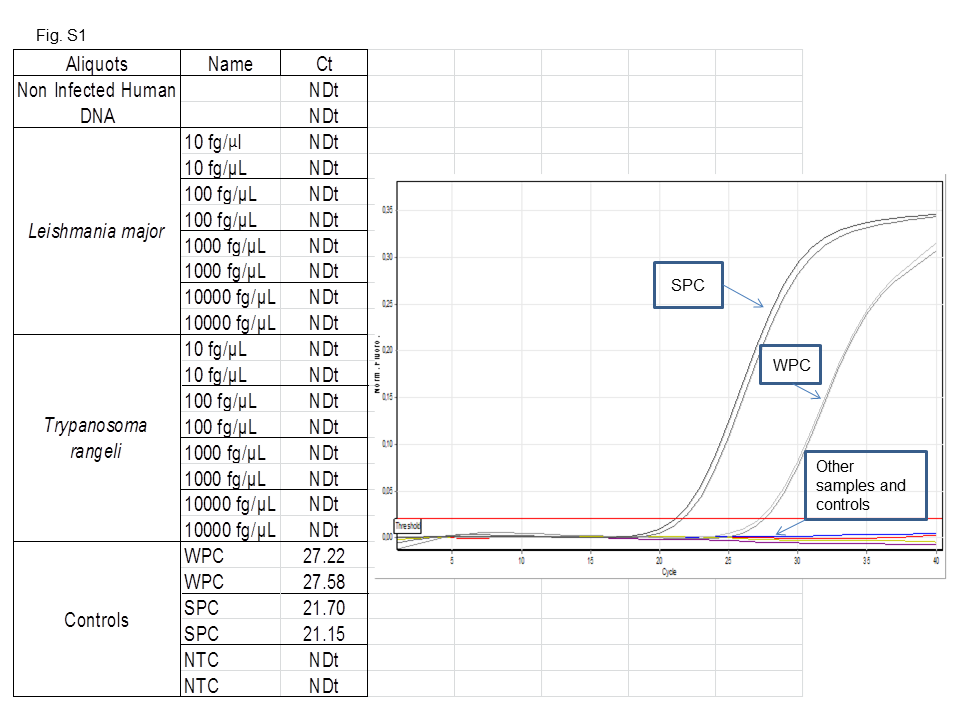

Supplement: S1 Fig — Y axis indicates normalized fluorescence and X axis denotes Cycles. SPC: Strong positive control: 10 fg/μL of T.cruzi DNA; WPC: weak positive control: 1 fg/μL of T.cruzi DNA. (TIF) [file pntd.0005779.s001.tif]

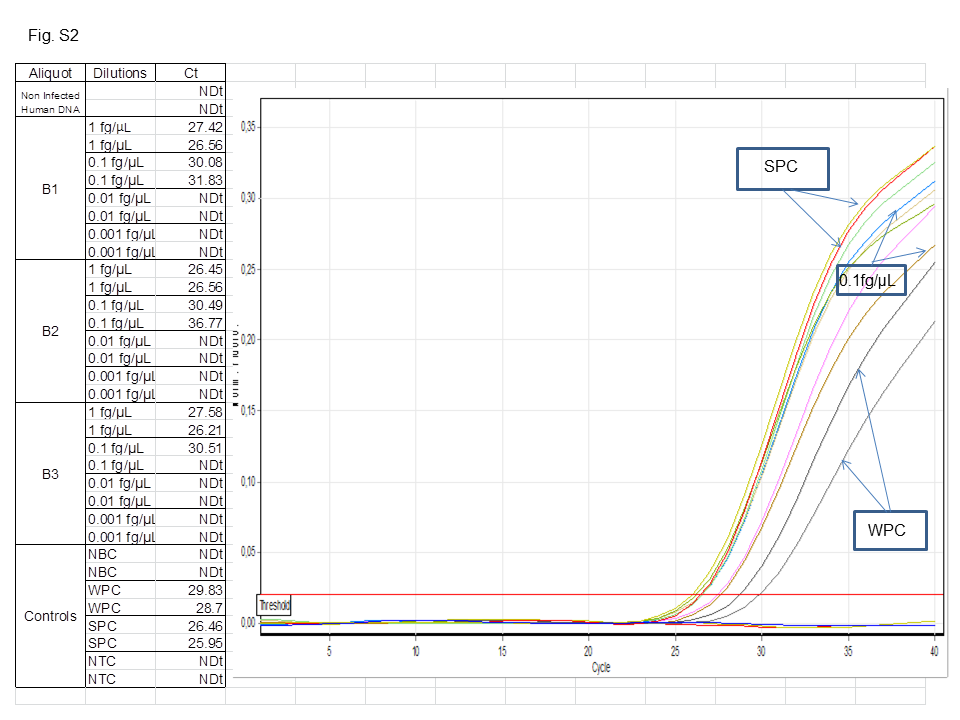

Supplement: S2 Fig — qPCR assays were performed using CL Brener DNA at concentrations ranging from 1 x 10^−3^ to 1 fg/μL. SPC: Strong positive control: 10 fg/μL of T.cruzi DNA; WPC: weak positive control: 1 fg/μL of T.cruzi DNA. Y axis indicates normalized fluorescence and X axis denotes Cycles. (TIF) [file pntd.0005779.s002.tif]

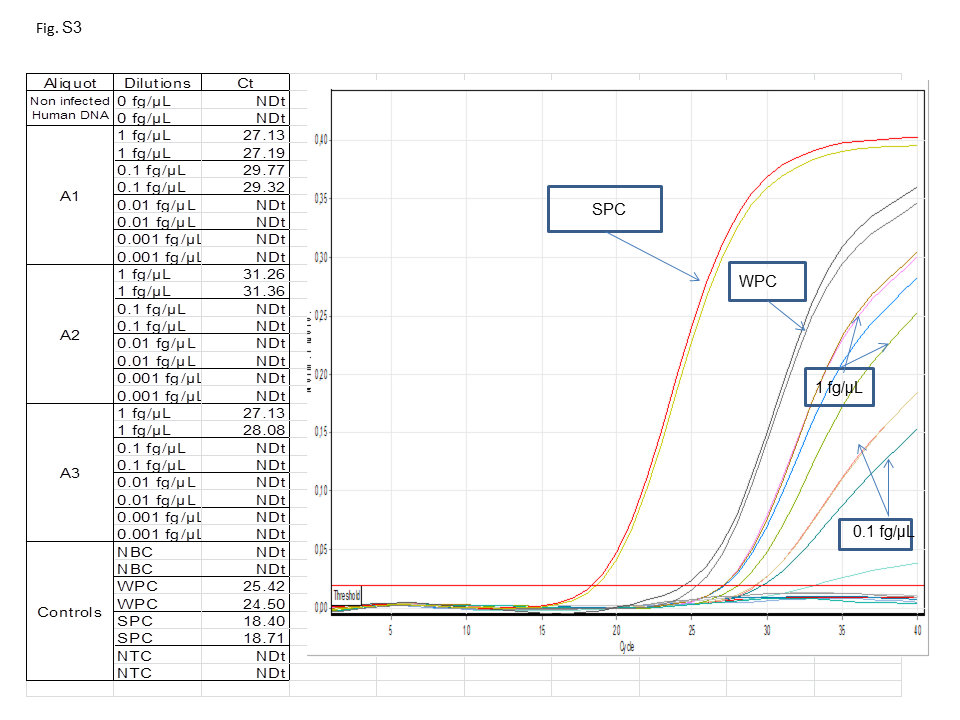

Supplement: S3 Fig — qPCR assays performed using the same concentrations of Sylvio X10 DNA tested by LAMP. SPC: Strong positive control: 10 fg/μL of T.cruzi DNA; WPC: weak positive control: 1 fg/μL of T.cruzi DNA. Y axis indicates normalized fluorescence and X axis denotes cycles. (TIF) [file pntd.0005779.s003.tif]

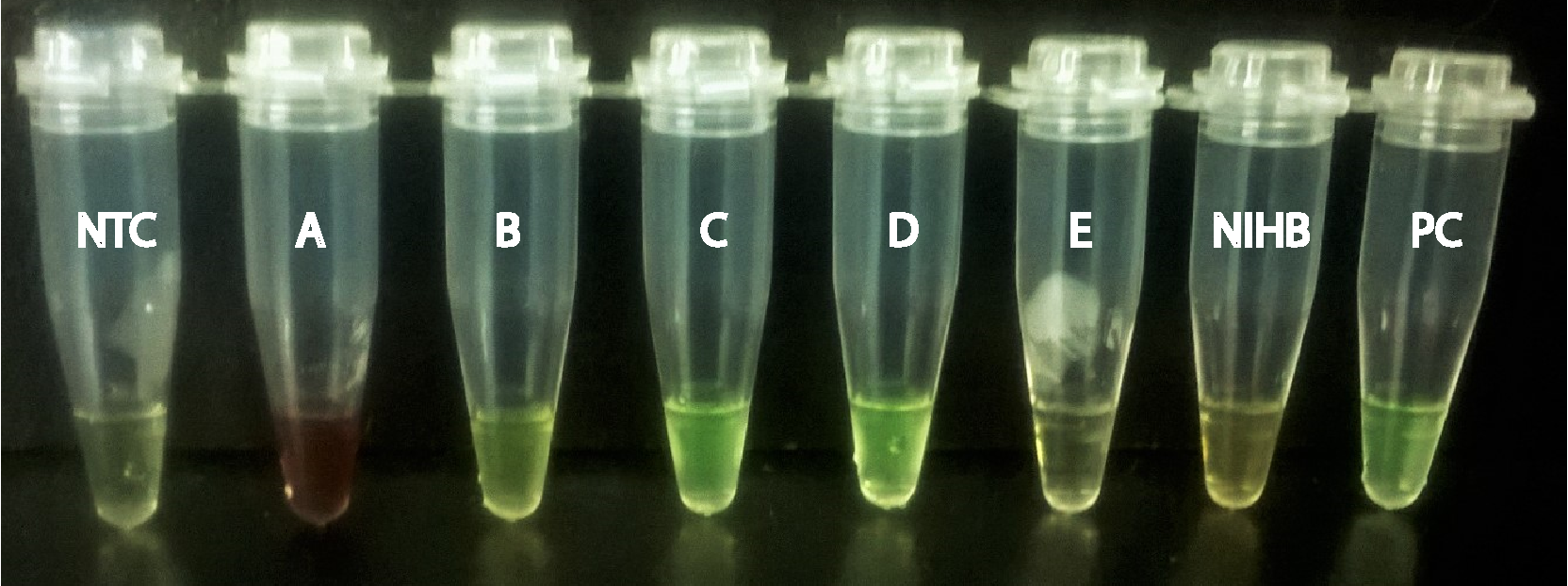

Supplement: S4 Fig — LAMP test of serial ten-fold dilutions of T.cruzi DNA extracted from a 104 par.eq./mL spiked heparinized blood sample using Boil & Spin method. NTC: Negative Control (distilled water); A, 1:10 dilution (10 3 par.eq/mL); B, 1:100 dilution (10 2 par.eq./mL); C, 1:1000 dilution(10 par.eq./mL); D,1:104 dilution (1 par.eq./mL); 1:105 dilution (E: 10-1 par.eq./mL); NIHB: Non Infected Human Blood; PC: Positive C. (TIF) [file pntd.0005779.s004.tif]

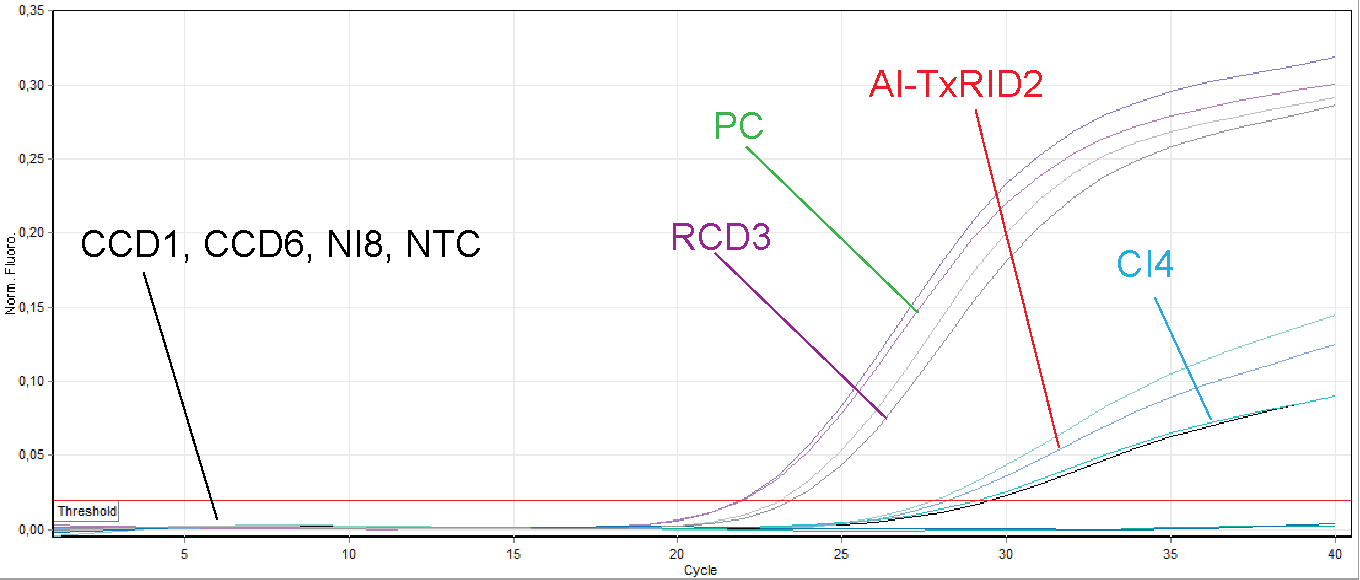

Supplement: S5 Fig — NTC: non template control; SPC: strong positive control; WPC: weak positive control. Clinical samples indicated in Fig 6, panel A. The Y axis denotes fluorescence and the X axis denotes Cts. control. (TIF) [file pntd.0005779.s005.tif]
