# Supplementary material for: Analytical sensitivity and specificity of a loop-mediated isothermal amplification (LAMP) kit prototype for detection of Trypanosoma cruzi DNA in human blood samples
Source: PLoS Negl Trop Dis. 2017 Jul 20;11(7):e0005779. doi: 10.1371/journal.pntd.0005779 (PMC5544240; doi:10.1371/journal.pntd.0005779)
Supplement: S1 Table — (DOCX) [file pntd.0005779.s006.docx]

| Sample  Parasite equivalents/ mL) | Cт-Cruzi | Cт-IAC |
| --- | --- | --- |
| NTC | ND | ND |
|  | ND | ND |
| 10^-3^ | ND | 21.97 |
|  | ND | 24.65 |
| 10^-2^ | 34.16 | 24.45 |
|  | 36.13 | 24.38 |
| 10^-1^ | 33.59 | 24.64 |
|  | ND | 24.53 |
| 1 | 28.84 | 24.00 |
|  | 28.29 | 23.93 |
| 10^1^ | 25.41 | 24.91 |
|  | 25.38 | 24.85 |
| 10^2^ | 22.69 | 24.45 |
|  | 22.42 | 24.74 |
| 10^3^ | 18.70 | 22.53 |
|  | 19.34 | 23.11 |
| BNC | ND | 24.23 |
|  | ND | 24.45 |
| WPC | 28.91 | 30.32 |
|  | 28.63 | 30.16 |
| SPC | 22.68 | 26.63 |
|  | 22.45 | 26.89 |

**S1 Table – Analytical Sensitivity of qPCR in EDTA blood samples spiked with serial dilutions of *T. cruzi* cells.**

**NTC**: Non template control, **BNC**: Seronegative Blood Control, **WPC** : Weak Positive control (0.2 fg/µL), **SPC**: Strong Positive Control (2 fg/µl).
